# PulmoCor: national registry for pulmonary hypertension

**DOI:** 10.1007/s12471-016-0830-x

**Published:** 2016-04-05

**Authors:** M. C. Post, A. P. Van Dijk, E. S. Hoendermis, H. J. Bogaard, V. Van Empel, K. A. Boomars

**Affiliations:** 1 Department of Cardiology, St. Antonius Hospital, Nieuwegein, the Netherlands; 2 Department of Cardiology, Radboud University Medical Center, Nijmegen, the Netherlands; 3 Department of Cardiology, University Medical Center Groningen, Groningen, the Netherlands; 4 Department of Pulmonary Diseases, VU University Medical Center, Amsterdam, the Netherlands; 5 Department of Cardiology, Maastricht University Medical Center, Maastricht, the Netherlands; 6 Department of Respiratory Medicine, Erasmus Medical Center, Rotterdam, the Netherlands

Pulmonary hypertension (PH) is a condition that refers to a mean pulmonary artery pressure of at least 25 mmHg measured by right heart catheterisation at rest. PH is classified in five groups based on the underlying clinical condition [1]. Some of these conditions are orphan diseases and managed in designed tertiary PH centres with dedicated multidisciplinary teams. During the last decades great progress has been made on the epidemiology, aetiology, pathophysiology and treatment of PH. Some of these efforts were mainly based on national PH registries, first published in the early 1980 s [2]. The primary goal of these clinical observational PH registries is to describe patients with PH and the impact of this disease [3]. Until recently, no such a national PH registry was available in the Netherlands.

In 2012, the Dutch multidisciplinary multicentre registry for PH (PulmoCor) was initiated by three tertiary PH centres and supported by the Interuniversity Cardiology Institute of the Netherlands (ICIN). Nowadays, six tertiary PH centres participate in this program (5 university medical centres and 1 non-university hospital). A working group including PH specialists (both cardiologists and pulmonologists) of all participating centres was established and regulations for this project have been drawn up. Several privacy-enhancing technologies have been used in accordance with the Dutch Privacy protections laws for data collection.

The aims of the PulmoCor registry are to further optimise patient care and research in the field of PH, and the joint collaboration between the national and international PH centres. Interestingly, the same structured web-based PH database, specially developed for the management of PH patients (PAHtool, Inovolutus, Portugal), is used in other international PH centres as well, both European and non-European [4]. The PulmoCor registry will facilitate investigation of the epidemiology of specific subtypes of PH, the treatment and long-term outcome. It will increase the awareness of this rare disease throughout the Netherlands. Furthermore, the registry can be used as clinical database as well.

Based on the current international guidelines, all PH centres used a standardised diagnostic approach for PH analysis [1]. Different data regarding the initial work-up and follow-up could be included in the PulmoCor registry. Each participating PH centre is using the secured uniformly structured database for the registration of their patients and these data are only accessible by this centre. To date, a total of 1493 patients with pulmonary hypertension (both prevalent and incident cases) have been included in the PulmoCor registry. In Fig. 1, this total group is shown according to the clinical classification (**a**) and the subgroup of patients with pulmonary arterial hypertension (PAH), group 1 (**b**).

**Fig. 1:**
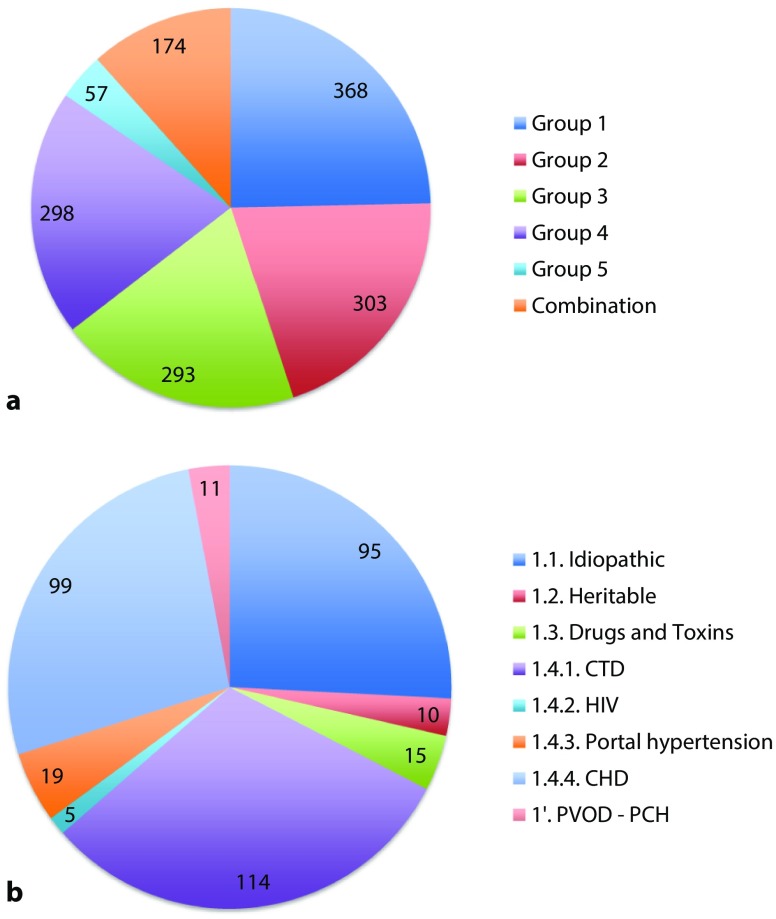
Total number of patients with pulmonary hypertension included in the PulmoCor registry divided by clinical classification (**a**) and the subgroup of pulmonary arterial hypertension (**b**). Group 1 Pulmonary arterial hypertension; Group 2 PH due to left heart disease; Group 3 PH due to lung disease and/or hypoxia; Group 4 Chronic thromboembolic PH and other pulmonary artery obstructions; Group 5 PH with unclear and/or multifactorial mechanisms [1]. (*CTD* connective tissue disease, *HIV* human immunodeficiency virus, *CHD* congenital heart disease, *PVOD* pulmonary veno-occlusive disease, *PCH* pulmonary capillary haemangiomatosis)

The uniformly designed PulmoCor registry data acquisition will increase the value of national collaboration both clinically and scientifically.

## References

[1] Galiè N, Humbert M, Vachiery J-L (2015). ESC/ERS Guidelines for the diagnosis and treatment of pulmonary hypertension. Eur Resp J.

[2] Awdish R, Definition CH (2015). epidemiology and registries of pulmonary hypertension. Heart Fail Rev.

[3] McGoon MD, Benza RL, Escribano-Subias P (2013). Pulmonary arterial hypertension: epidemiology and registries. J Am Coll Cardiol.

[4] Baptiste R, Meireles J, Agapito A (2013). Pulmonary hypertension in Portugal: first data from a nationwide registry. Biomed Res Int.

